# Spheroids of HER2-Positive Breast Adenocarcinoma for Studying Anticancer Immunotoxins In Vitro

**Published:** 2017

**Authors:** I. V. Balalaeva, E. A. Sokolova, A. D. Puzhikhina, A. A. Brilkina, S. M. Deyev

**Affiliations:** Lobachevsky State University of Nizhny Novgorod, Gagarin Ave., 23, Nizhny Novgorod, 603950, Russia; Shemyakin–Ovchinnikov Institute of Bioorganic Chemistry, Russian Academy of Sciences, Miklukho-Maklaya Str., 16/10, Moscow, 117997, Russia; National Research Tomsk Polytechnic University, Lenin Ave., 30, Tomsk, 634050, Russia

**Keywords:** 4D5scFv-PE40 immunotoxin, cancer marker HER2, drug penetration into tumor, spheroids, targeted therapy

## Abstract

Tumor response to therapeutic treatment is largely determined by its heterogeneity and the presence of intercellular junctions, hindering the penetration of large molecules deep into the three-dimensional structure of the tumor. In that context, 3D *in vitro *tumor models such as cancer cell spheroids are becoming increasingly popular. We obtained spheroids of human breast adenocarcinoma SKBR-3 overexpressing the HER2 cancer marker. The toxicity of HER2-targeted immunotoxin 4D5scFv-PE40 against spheroids was shown to be several orders of magnitude lower compared to a monolayer cell culture. The significant difference in the severity of the immunotoxin effect can be explained by the fact that it ineffectively penetrates the spheroid and predominantly influences the cells of the outer layers. The resulting tumor spheroid model can be used in development of drugs for targeted therapy as well as to study ways to improve the efficiency of anticancer agents by targeting cell–cell contacts.

## INTRODUCTION

The complex structure of solid tumors *in vivo *causes serious difficulties in *in vitro *studies of tumor development and when evaluating the therapeutic potential of anticancer drugs. A monolayer cell culture, despite its widest distribution, fails to reflect a number of features of the real tumor, especially its 3D organization. The 3D structure of a tumor implies numerous cell–cell contacts and considerable gradients of gases, nutrients, and catabolites throughout the tumor volume, resulting in the formation of a specific microenvironment for the cells of different layers. In turn, this leads to an heterogeneity of tumor cell populations that manifests itself as a variation of gene expression profiles and metabolism. A tumor’s heterogeneity largely determines its response to a therapeutic treatment. Furthermore, cell–cell contacts hinder the penetration of large molecules into the tumor, whereby the efficiency of drugs is strongly impacted by their ability to diffuse through the tumor mass [1]. In this regard, 3D *in vitro *tumor models, such as cancer cell spheroids, are becoming increasingly popular. Multicellular tumor spheroids are compact conglomerates of cancer cells that represent the avascular stage of tumor node development: a small primary tumor and early metastasis or the tumor zone located away from the vessel. The structural similarity between the spheroid and a real tumor increases the relevance of such a model, enabling a more accurate evaluation of potential anticancer agents under *in vitro *conditions [2, 3]. Taking into account the rapid development of targeted (directed) therapy [4], it is of particular interest to produce and employ cell spheroids that express the molecular targets that determine the specificity of a targeted agent.

We obtained spheroids of human breast adenocarcinoma SKBR-3 overexpressing the HER2 cancer marker and demonstrated that this model is informative in the assessment of the penetration depth and anticancer efficiency of HER2-targeted immunotoxin 4D5scFv- PE40.

## EXPERIMENTAL

**Production of spheroids of human breast adenocarcinoma**

HER2-overexpressing human breast adenocarcinoma cells, SKBR-3 (ATCC number HTB-30), were used [5]. The cells were cultured in a McCoy’s 5A medium with 1.5 mM L-glutamine (HyClone, USA) supplemented with 10% (v/v) fetal calf serum (HyClone, USA) at 37°C in 5% CO_2_. For passaging, the cells were carefully detached using a Versene solution (PanEco, Russia).

The spheroids were produced following three protocols. For the first one, we used 96-well culture plates with the standard adhesive surface (Corning, USA) pre-coated with 1% agarose (AppliChem, Germany) in distilled water (50 μl per well). For the second and third protocols, we used 96-well Ultra-Low-Attachment Microplates (Corning, USA) with a round or flat bottom, respectively. In all cases 200 cells were seeded per well.

The images of the spheroids were captured by phase contrast microscopy on an Axiovert 200 inverted microscope with an EC Plan-Neofluar 10 × /0.3 objective lens (Carl Zeiss, Germany). The volume of a spheroid (V, mm3) was calculated according to the equation: *V *= *a*×*b*2 ⁄2, where *a *is the larger diameter (μ) and *b *is the smaller diameter (μ).

**Production of 4D5scFv-PE40 immunotoxin**

Recombinant immunotoxin 4D5scFv-PE40 [6] was produced in *Escherichia coli *cells, strain BL21(DE3), transformed with the plasmid pSD-4D5scFv-PE40 containing the gene of the protein under *lac*-promoter control. The protein was purified successively by metal-chelate affinity chromatography using a HisTrap FF 1 ml column (GE Healthcare, USA) and ion exchange chromatography on a QSepharose FF 1 ml column (GE Healthcare, USA) according to the manufacturer’s instructions. Fractions containing the desired protein were analyzed by electrophoresis in 12% PAG under denaturing conditions according to the standard protocol.

**Analysis of the cytotoxicity of 4D5scFv-PE40 immunotoxin against a SKBR-3 monolayer culture and spheroids**

For the cytotoxicity study on a monolayer culture, the SKBR-3 cells were seeded in a 96-well plate (Corning, USA), 2,000 cells per well, and grown overnight. The medium was then replaced with a fresh one containing 4D5scFv-PE40 at various concentrations (10-5–102 nM), and the cells were incubated for 72 h. Cell viability was estimated using a MTT assay [7]. The medium was replaced with a fresh one containing 0.5 mg/ml MTT (Alfa Aesar, Great Britain), followed by incubation for 4 h. The formed formazan crystals were solved in DMSO (PanEco, Russia), and the optical density at 570 nm was measured on a Synergy MX microplate reader (BioTek, USA). The spheroids were produced as described above using 96-well round bottom ultra-low-attachment plates and cultured overnight. The cytotoxicity of the 4D5scFv-PE40 immunotoxin against the spheroids was studied in the same way with the incubation time increased up to 168 h.

Relative cell viability was represented as a percentage of the average optical density in the wells with treated cells to the average optical density in the wells with untreated cells. The cytotoxicity of the immunotoxin against the spheroids was also evaluated according to a spheroid’s volume on the final day of incubation in the presence of immunotoxin: relative cell viability was in this case calculated as a percentage of the mean volumes of treated to untreated spheroids. Data were processed using the GraphPad Prism 6 software (GraphPad Software). The IC_50_ was calculated by nonlinear regression using the four-parameter dose-response model.

**Assessment of immunotoxin 4D5scFv-PE40 penetration of spheroids**

In order to visualize the penetration of 4D5scFv-PE40 into a spheroid, the immunotoxin was conjugated with a low-molecular-weight fluorescent dye, DyLight650. For the reaction, the protein was exchanged into borate buffer (400 mM H_3_BO_3_, 70 mM Na_2_B_4_O_7_, pH 8.0) by gel filtration on a PD SpinTrap G-25 column (GE Healthcare, USA). N-Hydroxysuccinimide derivative DyLight650 NHS Ester (Thermo Fisher Scientific, USA) that ensures dye conjugation to the protein primary amino groups was used. The protein was incubated with a sevenfold molar excess of DyLight650 NHS Ester diluted in DMSO for 1 h at room temperature in the dark in accordance with the manufacturer’s recommendations. Unbound dye was removed by gel filtration on a PD SpinTrap G-25 column equilibrated with phosphate buffered saline (PBS), pH 7.4 (PanEco, Russia).

SKBR-3 cells were seeded in a 96-well ultra-low-attachment round-bottom plate, 1,000 cells per well, and grown overnight to produce spheroids. The formed spheroids were incubated in a medium containing fluorescent conjugates of 4D5scFv-PE40 for 2 h at 37°C. The spheroids were then washed twice with PBS and fixed with 10% formalin in PBS for 15 min in the dark. The images of the spheroids were obtained using an Axio Observer Z1 LSM 710 NLO/Duo confocal microscope (Carl Zeiss, Germany) equipped with a EC Plan- Neofluar 20×/0.50 objective lens. DyLight650 fluorescence was excited at 633 nm with a helium-neon laser. The signal was registered in the range of 643–735 nm.

## RESULTS AND DISCUSSION

Cancer cells, including a number of human breast cancer cell lines, are generally known to show a strong tendency toward forming spheroids in a culture [8-12]. However, the difficulties associated with obtaining well-shaped cell spheroids of SKBR-3 cells were encountered in several studies in a wide range of culture conditions, including when extracellular matrix components (Matrigel) were added into the growth medium or the medium viscosity was increased by adding methylcellulose [9, 10].

**Fig. 1:**
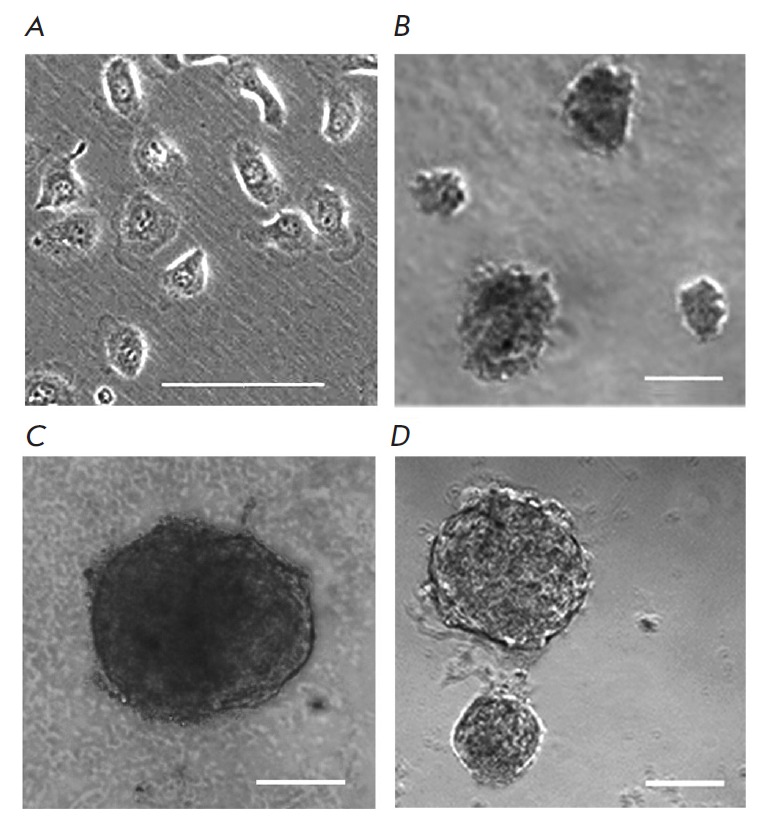
Morphology of SKBR-3 cells in a monolayer culture (A) and after culturing for 8 days under non-attachment conditions: agarose (B) and ultra-low attachment plates with round (C) or flat (D) bottom. Bar, 50 μm (A) or 100 μm (B, C, D).

Culturing SKBR-3 cells under various conditions that prevent the formation of a monolayer culture (*Fig. 1A*) revealed their significant impact on the spheroid-formation ability of a cell culture. The cells grown on agarose formed loose aggregates of irregular shape that greatly varied in size and had jagged edges. The diameter of the largest aggregates was 30–100 μm on the 2nd day after cell seeding and reached 60–140 μm within 8 days of culturing (*Fig. 1B*).

Utilization of the ultra-low-attachment plates was more successful. A single spheroid was formed in each well of the round-bottom plate after culturing for one day; it was a tight round-shaped conglomerate of cells with a diameter of about 160–200 μm and a clearly defined edge. By day 8 of culturing, the spheroids had reached 250–560 μm in diameter (*Fig. 1C*). When cultured in flat-bottom plates, 20–30 round-shaped spheroids were formed per well, mostly with a clear smooth edge and size of 30–60 and 130–360 μm on culturing days 2 and 8, respectively (*Fig. 1D*).

**Fig. 2:**
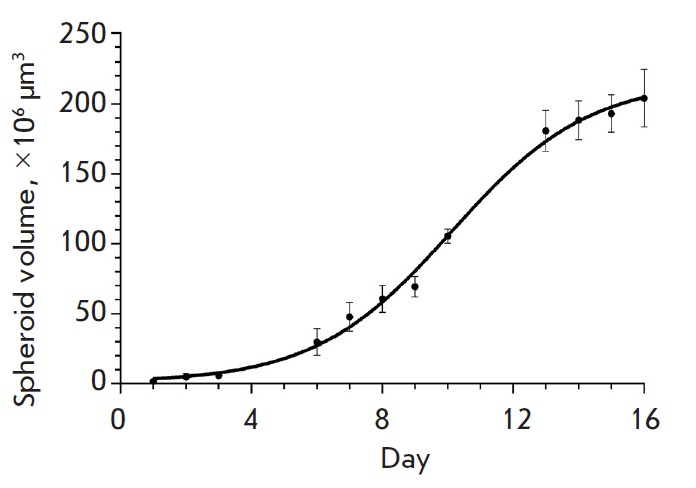
The growth curve of SKBR-3 spheroids in ultra-low attachment round-bottom plates. Mean ± SEM, n = 5. The cells were seeded in the plates on day 0.

With allowance for the features of formation of the 3D structures by SKBR-3 cells under various culturing conditions, the round-bottom ultra-low-attachment plate was acknowledged as the optimal one and was further used. The growth dynamics of the spheroids produced using this technique was shown to be complex: the initial exponential phase that lasted for about 10 days in our experiment gave way to a phase of slower growth (*Fig. 2*). A similar behavior described by the Gompertz function characterizes tumor growth *in vivo* [13]. The deceleration in spheroid growth can be attributed to the change in the ratio between different cell populations as its size rises and to the increasingly hindered supply of oxygen and nutrients deep into the spheroid: the increase in the proportion of non-dividing (resting) cells and/or death of resting cells accompanied by enlargement of the necrotic core [3, 4].

Overexpression of the HER2 receptor is one of the key features of the SKBR-3 cell line used for spheroid production. This receptor belongs to the epidermal growth factor receptor family and is an important component of the signal transduction network that controls cell proliferation, differentiation, and apoptosis [15]. The high level of HER2 expression that is typical of many types of tumors and its role in tumor pathogenesis make this receptor an advanced target for targeted anticancer drugs [16, 17]. Recombinant immunotoxins, fusion proteins comprising functionally independent targeting and toxic modules, are promising agents for targeted therapy. Antibody fragments or non-immunoglobulin polypeptides act as targeting modules that provide directed delivery of such molecular constructs to cancer cells, while the toxic effect is ensured by naturally modified toxin proteins of various origins [18]. We analyzed the growth of spheroids under the influence of the previously created recombinant immunotoxin 4D5scFv-PE40 that comprised HER2-specific antibody 4D5scFv as a targeting module and a 40 kDa fragment of *Pseudomonas *exotoxin A (PE40) as a toxic module [6].

**Fig. 3:**
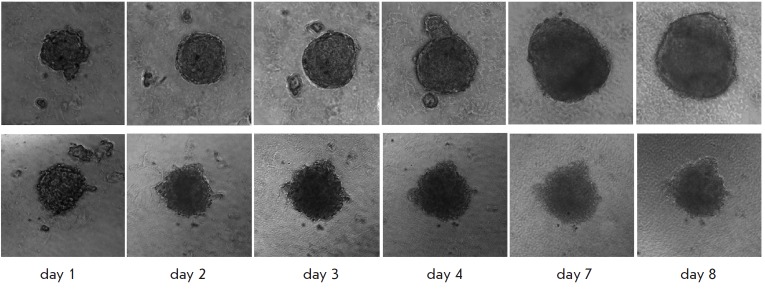
Morphology of SKBR-3 spheroids on different days of growth in the control (*upper panel*) and in the presence of 100 nM 4D5scFv-PE40 (*lower panel*). Image size 400×400 μm.

**Fig. 4:**
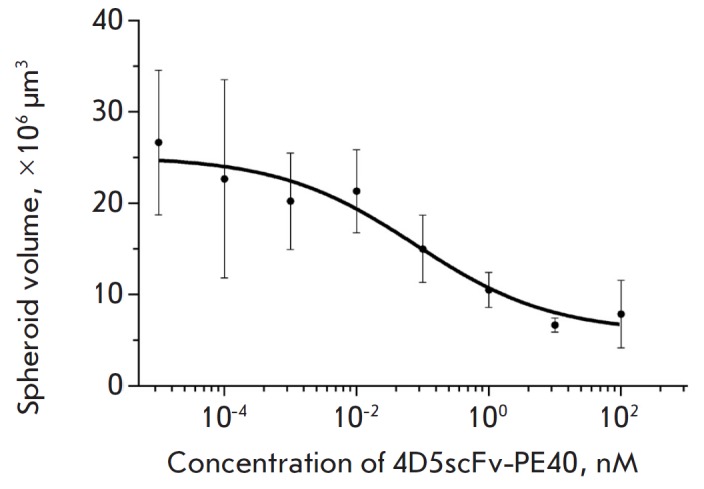
Size of SKBR-3 spheroids after incubation for 7 days in the presence of 4D5scFv-PE40 at different concentrations. Mean ± SEM, n = 6 for all experimental conditions.

The presence of 4D5scFv-PE40 in the growth medium significantly slowed down spheroid growth (*Fig. 3*). The impact of the immunotoxin on the spheroid size was dose-dependent (*Fig. 4*). Complete inhibition of spheroid growth was achieved at immunotoxin concentrations higher than 1 nM (*Fig. 3*, lower row). The immunotoxin also affected the spheroid morphology: in contrast to the control, the spheroids loosened and lost their characteristic shape as early as on day 2 of incubation with the immunotoxin (*Fig. 3*, lower row). Tight packing of cells in the spheroid structure is known to be made possible by an increased expression of cell junction proteins, in particular cadherins, and their accumulation on the cell surface [19, 20]. Since the toxic effect of *Pseudomonas *exotoxin A is a result of the blockage of protein synthesis in target cells [21], the observed effect of 4D5scFv-PE40 immunotoxin on the spheroid morphology may be due to a reduction in the amount of cell adhesion proteins in the cells.

**Fig. 5:**
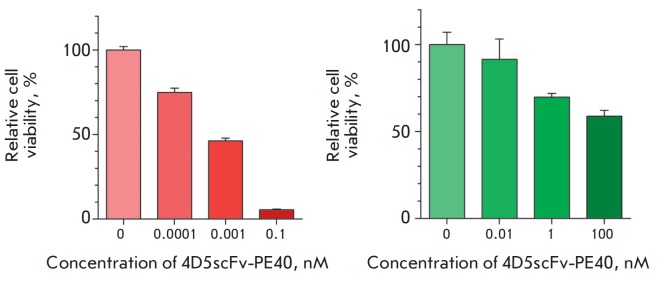
Effect of 4D5scFv-PE40 on SKBR-3 culture growth. *Left-hand side: *the relative viability of the monolayer culture after incubation with immunotoxin for 72 h. *Right-hand side: *the relative viability of the spheroid cells 168 h after the immunotoxin was added into the medium. Mean ±SEM, n = 6 for all experimental conditions.

A comparative analysis of 4D5scFv-PE40 cytotoxicity against SKBR-3 cells in the monolayer and spheroids estimated by a MTT assay showed significant resistance by the spheroids to this agent. Thus, the effect of 4D5scFv-PE40 against the monolayer culture was observed at concentrations ranging from 0.1 pM to 0.1 nM with IC_50_ of about 0.8 pM after 72 h of incubation (*Fig. 5*, left). This is consistent with the results obtained earlier for another HER2-overexpressing cell line, SKOV-3 [22]. However, the viability of the cells in the spheroids practically did not decrease under the same conditions. When the incubation time was increased to 168 h, the toxic effect of 4D5scFv-PE40 immunotoxin was observed only when its concentration was increased to 1 nM and IC_50_ was higher than 100 nM (*Fig. 5*, right).

The phenomenon of greater resistance shown by tumors *in vivo *to therapeutic agents compared to corresponding cancer cells in culture is well known [23, 24]. We have previously shown the toxic effect of the 4D5scFv-PE40 immunotoxin at picomolar concentrations on the HER2-overexpressing human ovarian adenocarcinoma cells SKOV-kat in culture, while its *in vivo *activity against SKOV-kat xenograft tumors becomes evident under its administration at nanomolar concentration [25]. The use of a monolayer culture of cancer cells obviously does not allow one to predict the effective range of concentrations of a tested agent in the whole organism.

Resistance usually depends on a combination of factors different in nature that represent both the tumor properties and the pharmacokinetics of the drug. One of these factors is insufficient drug accumulation in the tumor as a result of its poor penetration into the tumor mass, which in turn can arise from high interstitial fluid pressure, irregular arrangement of tumor blood vessels, numerous intercellular contacts, and/or the presence of extracellular matrix components. This is of particular importance for protein drugs: notably, recombinant immunotoxins, whose molecule size (50– 70 kDa) results in a short blood circulation time (20–30 min) on the one hand, while on the other hand it slows down diffusion into tissues [26-28].

**Fig. 6:**
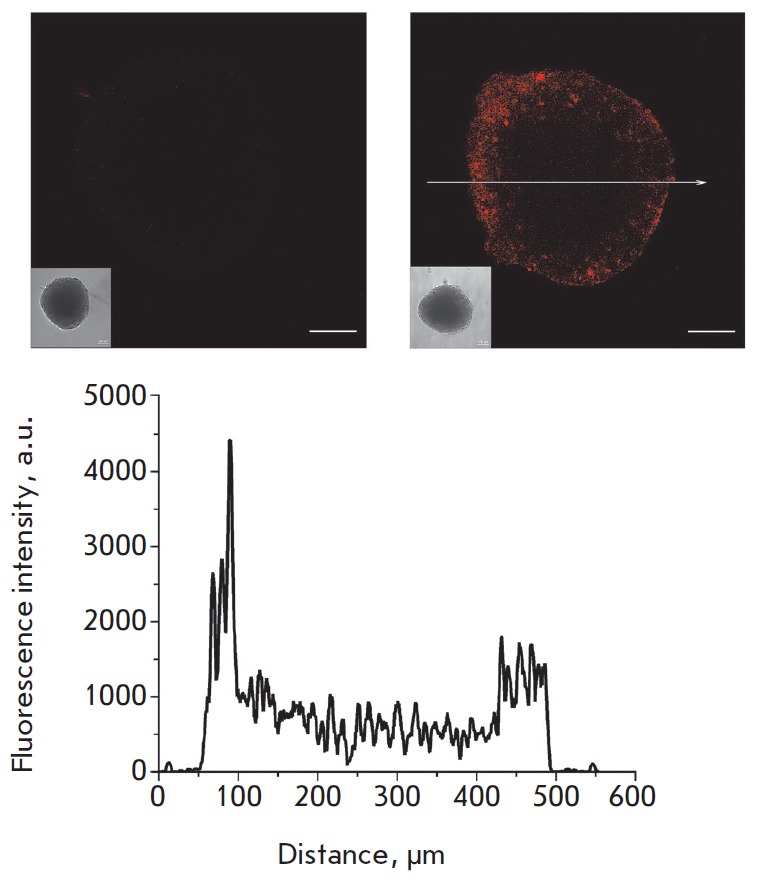
Confocal images of an unstained SKBR-3 spheroid (*left*) and SKBR-3 spheroid stained for 2 h with 4D5scFv- PE40 conjugated with a DyLight650 fluorescent dye (*right*). Bar, 100 μm. The insets show wide-field microscopy images of the same spheroids. Bottom: the fluorescence signal profile along the arrow shown in the right-hand side image.

In order to estimate the depth of the immunotoxin penetration into the spheroid, we conjugated 4D5scFv- PE40 with the DyLight650 fluorescent dye. We discovered that after incubation for 2 h, the 4D5scFv- PE40 immunotoxin labeled with the fluorescent dye had penetrated to a depth of about 80–100 μm (into a spheroid 400–500 μm in diameter), which corresponds to several surface cellular layers (*Fig. 6*). These results are consistent with the data on the penetration of antibody Fab-fragments with a size lying in the same range (about 50 kDa) into the spheroids of human colon cancer [29].

## CONCLUSION

Thus, the significant difference between the severity of the toxic effect of the 4D5scFv-PE40 immunotoxin on spheroids and on a monolayer of SKBR-3 cells can largely be attributed to its inefficient penetration into the depth of the spheroid and to its predominant impact on the cells of the outer layers. In this case, the use of spheroids consisting of tumor cells only enables an evaluation of the direct influence of cell–cell contacts on the test drug efficiency. In this regard, we believe that the obtained tumor spheroid model can be successfully used to study ways to improve the efficiency of accumulation of anticancer agents in a tumor via the simultaneous influence on cell–cell contacts. This is of particular interest for designing and testing HER2-specific agents, since the HER2 receptor is usually hidden under cell adhesion proteins and may be unavailable for binding by the targeted agent [30, 31]. Such influence is possible for proteins that target intercellular tight junctions [32]. This approach was proposed several years ago and has proved to be effective when full-length therapeutic antibodies are used and appears to be of interest for the development of targeted cancer therapy.

## Abbreviations

HER2: human epidermal growth factor receptor 2
scFv: single-chain variable fragment
PE40: 40 kDa fragment of Pseudomonas exotoxin A
DMSO: dimethyl sulfoxide
MTT: 3-(4,5-dimethylthiazol-2-yl)-2,5-diphenyltetrazolium bromide
PAG: polyacrylamide gel

## References

[1] Minchinton A.I., Tannock I.F. (2006). Nat. Rev. Cancer..

[2] Antoni D., Burckel H., Josset E., Noel G. (2015). Int. J. Mol. Sci..

[3] Weiswald L.B., Bellet D., Dangles-Marie V. (2015). Neoplasia..

[4] Deyev S.M., Lebedenko E.N., Petrovskaya L.E., Dolgikh D.A., Gabibov A.G., Kirpichnikov M.P. (2015). Russ. Chem. Rev..

[5] Hynes N.E., Gerber H.A., Saurer S., Groner B. (1989). J. Cell Biochem..

[6] Sokolova E.A., Zdobnova T.A., Stremovskiy O.A., Balalaeva I.V., Deyev S.M. (2014). Biochemistry (Mosc.)..

[7] Mosmann T. (1983). J. Immunol. Meth..

[8] Glinsky V.V., Huflejt M.E., Glinsky G.V., Deutscher S.L., Quinn T.P. (2000). Cancer Research.

[9] Ivascu A., Kubbies M. (2007). Int. J. Oncol..

[10] Froehlich K., Haeger J.D., Heger J., Pastuschek J., Photini S.M., Yan Y., Lupp A., Pfarrer C., Mrowka R., Schleussner E., Markert U.R., Schmidt A. (2016). J. Mammary Gland Biol. Neoplasia..

[11] Akasov R., Haq S., Haxho F., Samuel V., Burov S.V., Markvicheva E., Neufeld R.J., Szewczuk M.R. (2016). Oncotarget..

[12] Debnath J., Brugge J.S. (2005). Nat. Rev. Cancer..

[13] Hjelstuen M.H., Rasch-Halvorsen K., Brekken C., Bruland O. (1996). Acta Oncol..

[14] Lin R.Z., Chang H.Y. (2008). Biotechnol. J..

[15] Polanovski O.L., Lebedenko E.N., Deyev S.M. (2012). Biochemistry..

[16] Harari D., Yarden Y. (2000). Oncogene..

[17] Yan M., Parker B.A., Schwab R., Kurzrock R. (2014). Cancer Treat Rev..

[18] Kreitman R.J. (2006). Aaps J..

[19] Mueller S., Cadenas E., Schonthal A.H. (2000). Cancer Research.

[20] Xiang X., Phung Y., Feng M., Nagashima K., Zhang J., Broaddus V.C., Hassan R., Fitzgerald D., Ho M. (2011). PLoS One..

[21] Weldon J.E., Pastan I. (2011). FEBS J..

[22] Sokolova E.A., Stremovskiy O.A., Zdobnova T.A., Balalaeva I.V., Deyev S.M. (2015). Acta Naturae..

[23] Niero E.L., Rocha-Sales B., Lauand C., Cortez B.A., de Souza M.M., Rezende-Teixeira P., Urabayashi M.S., Martens A.A., Neves J.H., Machado-Santelli G.M. (2014). J. Exp. Clin. Cancer Res..

[24] Fong E.L., Harrington D.A., Farach-Carson M.C., Yu H. (2016). Biomaterials..

[25] Zdobnova T., Sokolova E., Stremovskiy O., Karpenko D., Telford W., Turchin I., Balalaeva I., Deyev S. (2015). Oncotarget..

[26] Weldon J.E., Xiang L., Zhang J., Beers R., Walker D.A., Onda M., Hassan R., Pastan I. (2013). Mol. Cancer Ther..

[27] Zielinski R., Lyakhov I., Hassan M., Kuban M., Shafer-Weaver K., Gandjbakhche A., Capala J. (2011). Clin. Cancer Res..

[28] Cao Y., Marks J.W., Liu Z., Cheung L.H., Hittelman W.N., Rosenblum M.G. (2014). Oncogene..

[29] Sutherland R., Buchegger F., Schreyer M., Vacca A., Mach J.P. (1987). Cancer Research.

[30] Choi I.K., Strauss R., Richter M., Yun C.O., Lieber A. (2013). Front. Oncol..

[31] Beyer I., van Rensburg R., Lieber A. (2013). Tissue Barriers..

[32] Beyer I., van Rensburg R., Strauss R., Li Z., Wang H., Persson J., Yumul R., Feng Q., Song H., Bartek J., Fender P., Lieber A. (2011). Cancer Research.

